# Post-disaster psychological effects: identifying earthquake-induced trauma in athletes

**DOI:** 10.3389/fpsyg.2026.1804335

**Published:** 2026-06-23

**Authors:** Fatih Mehmet Uğurlu, Muhammed Güler, Veysel Albayrak, Caner Aygören

**Affiliations:** 1 Faculty of Sport Sciences, Firat Universitesi, Elâzığ, Türkiye; 2 Faculty of Sport Sciences, Munzur University, Tunceli, Türkiye

**Keywords:** athlete, earthquake trauma, sport, sports psychology, trauma

## Abstract

Natural disasters not only cause physical destruction but also lead to various psychological problems affecting the mental health of individuals. This study was conducted to determine the levels of post-earthquake trauma in athletes who experienced earthquakes and whether certain variables made a difference in this situation. The sample of the study consisted of 359 athletes living in 11 provinces affected by the earthquakes centered in Kahramanmaraş, Turkey, on February 6, 2023. Participants were reached using the Snowball Sampling Method. The Sociodemographic Information Form and the Post-Earthquake Trauma Level Determination Scale were used to collect data. As a result of the descriptive statistics, it was seen that athletes experienced above-average earthquake trauma (M = 65.84). When examined in terms of sociodemographic variables, it was found that women showed more symptoms of earthquake trauma than men, those who experienced the loss of relatives showed more symptoms than those who did not, and those who experienced the loss of a home showed more symptoms than those who did not. In conclusion, while exercise and physical activity are methods used to cope with trauma, being an athlete alone is not sufficient to cope with earthquake trauma. Women, whether athletes or not, experience more earthquake trauma than men. Losing relatives exacerbates the trauma caused by the earthquake. Furthermore, the loss of a home, which prevents individuals from meeting some basic physiological needs, is a significant factor in earthquake trauma.

## 1 Introduction

Natural disasters like earthquakes can cause not only physical devastation to survivors but also long-lasting and profound psychological consequences. The resulting outcomes are among the most devastating traumatic events (Norris et al., 2002). The cognitive, affective, behavioral, and social effects of such disasters on people can persist for long periods (Uğuz, 2023). The most common psychological responses observed after an earthquake are post-traumatic stress disorder (PTSD) and an increase in trauma symptoms (Goldmann and Galea, 2014). The frequency of post-earthquake trauma symptoms is assessed in terms of individual and societal impacts (Acierno et al., 2006). The magnitude of this impact can vary depending on various characteristics such as the level of exposure and the time of assessment (Liang et al., 2021; Hosseinnejad et al., 2022). Previous studies have mentioned the existence of psychological effects such as anxiety, emotional distress, sleep disorders, and post-traumatic stress disorder after earthquakes (Cénat et al., 2020; Aker and Önder, 2025). Similarly, limited studies in athlete samples show that trauma levels can vary depending on different variables (Atılgan and Tükel, 2021).

On February 6, 2023, Türkiye experienced two catastrophic earthquakes measuring 7.8 and 7.6 in magnitude, which caused widespread destruction across 11 provinces. The earthquakes resulted in substantial loss of life and severe structural damage. Official reports indicated that more than 50,000 people lost their lives and hundreds of thousands of buildings were either destroyed or heavily damaged following the disaster (Republic of Türkiye Presidency of Strategy and Budget, 2023). Even after the devastating effects of major earthquakes subside, trauma-specific symptoms can emerge in individuals. These symptoms can negatively impact an individual’s daily life and quality of life (Sirotich and Camisasca, 2024). A meta-analysis of 46 studies found that the prevalence of post-traumatic stress disorder (PTSD) resulting from earthquakes was 23.66% (Dai et al., 2016). Furthermore, the onset of PTSD varies depending on factors such as gender, loss of loved ones, and damage to the home. These findings demonstrate that post-earthquake trauma has a multidimensional structure (Toper et al., 2025).

Moreover, the existing literature has largely focused on the psychological effects of earthquakes in the general population, while the relationship between sports participation and post-earthquake traumatic symptoms among athletes has received relatively limited attention. In this respect, the present study aims to contribute to the literature by examining post-earthquake trauma levels specifically in an athlete sample and by evaluating the role of sports participation on traumatic symptoms in the context of certain variables. Therefore, the study is expected to provide both theoretical contributions to sports psychology literature and practical implications for sports-based psychosocial intervention programs.

Study Questions:

1. What are the levels of trauma among athletes after the earthquake?
2. Do the trauma levels of athletes differ according to gender, loss of relatives in the earthquake, and loss of home?

## 2 Method

### 2.1 Research model

This study used the correlational survey model, one of the quantitative research methods. The correlational survey model is a research model that aims to determine the degree of covariation (relationship) between two or more variables (Karasar, 2012).

### 2.2 Research group

The population of this study consists of active licensed athletes living in 11 provinces affected and devastated by the earthquakes centered in Kahramanmaraş, Turkey, on February 6, 2023. The sample of the study consists of a total of 359 athletes reached using the snowball sampling method, a non-probability sampling method. The main reason for choosing this method is the difficulty in reaching athletes affected by the disaster in the aftermath of the earthquake, and the limited accessibility of the target group. Participants were reached through referrals from individuals included in the study. The study data was collected in the second year after the disaster.

### 2.3 Data collection tools

The Sociodemographic Information Form and the Post-Earthquake Trauma Level Assessment Scale were used as data collection tools in this study.

#### 2.3.1 Sociodemographic information form

The sociodemographic information form created by the researchers consisted of 3 questions. These variables included gender, experience of losing a relative, and experience of home loss.

#### 2.3.2 Post-Earthquake Trauma Level Assessment Scale

This study used the Earthquake Trauma Level Assessment Scale developed by Tanhan and Kayri (2013). The scale, developed to assess post-earthquake traumatic symptoms, consists of 20 items, 5 factors, and a 5-point Likert scale. High scores on the scale indicate high levels of post-earthquake trauma in individuals. The scale development study was conducted on 1,505 individuals affected by the Van earthquake. Exploratory and confirmatory factor analyses determined that the five-factor structure of the scale is valid. It was reported that the scale explains 54.29% of the total variance, with a KMO value of 0.917 and a significant Bartlett’s Sphericity Test result (χ^2^ = 7816.483, *p* < 0.01). Furthermore, the fit indices were found to be RMSEA = 0.000, NFI = 0.88, GFI = 0.94, RMR = 0.080, and AGFI = 0.92. The total Cronbach’s Alpha internal consistency coefficient of the scale was reported as 0.87. In this study, the total Cronbach’s Alpha coefficient of the scale was (0.923).

### 2.4 Data analysis

IBM SPSS Statistics 24 software package was used to analyze the study data. During the analysis, Cronbach’s alpha coefficients were calculated to determine the reliability of the scale, and the normality of the data distribution was evaluated by examining skewness and kurtosis values. In addition, the overall mean scores and frequency values of the participants were calculated; and the Independent Samples t-test was used to determine the differences between two groups.

## 3 Findings

An examination of the sociodemographic characteristics of the athletes participating in the study revealed that 189 were female and 170 were male. It was determined that 109 participants had lost relatives, while 250 had not. Furthermore, it was found that 92 participants had their homes destroyed in the earthquake, while 267 had their homes intact (Tables 1, 2).

**Table 1:** Sociodemographic variables.

Variable	Group	*n*	%
Gender	Female	189	52.6
	Male	170	47.4
Situation of experiencing loss of a relative	Yes	109	30.4
	No	250	69.6
Home loss situation	Yes	92	25.6
	No	267	74.4

A total of 359 athletes participated in the study.

**Table 2:** Overall mean scores, normality and reliability analysis results for the Post-Earthquake Trauma Level Assessment Scale.

Factor	Skewness	Kurtosis	Cronbach’s alpha	Min.	Max.	M	SD
Behavior problems	0.160	−0.872	0.780	4.00	20.00	11.17	4.42
Emotive limitedness	−0.013	−1.114	0.846	5.00	25.00	15.36	5.91
Affective	−0.103	−0.424	0.530	4.00	20.00	13.62	3.45
Cognitive structures	−0.739	0.052	0.742	4.00	20.00	15.39	3.83
Sleep problems	−0.435	−0.904	0.885	3.00	15.00	10.28	3.78
Total scale	−0.147	−0.809	0.923	21.00	100.00	65.84	17.86

Whether the study data showed a normal distribution was evaluated by examining the skewness and kurtosis values. The obtained values were found to be in the range of −1.5 to +1.5. Accordingly, it was decided to use parametric tests in the study (Tabachnick and Fidell, 2013).

Reliability analysis of the scales and their sub-dimensions revealed that Cronbach’s alpha coefficients were at a sufficient level (Nunnally and Bernstein, 1994).

When examining the findings of this study conducted on athletes who experienced an earthquake, it was observed that the behavioral problems factor (M = 11.17) was slightly below average according to the mean scores obtained from the Post-Earthquake Trauma Level Assessment Scale. While the emotive limitedness factor (M = 15.36) was determined to be at an average level, the affective factor (M = 13.62), the cognitive structures factor (M = 15.39), and the sleep problems factor (M = 10.28) were found to have values above average. The total mean score of the scale (M = 65.84) was determined to be above the scale average.

Table 3 presents the results of the independent samples *t-*test conducted according to sociodemographic variables.

**Table 3:** Independent samples *t*-test results for total scores and sub-factors of the Post-Earthquake Trauma Level Determination Scale according to variables.

Variables		Behavior problems		Emotive limitedness		Affective		Cognitive structures		Sleep problems		Total scale	
		M	SD	M	SD	M	SD	M	SD	M	SD	M	SD
Gender	Female	11.61	4.32	16.00	5.90	14.20	3.34	16.13	3.43	10.88	3.54	68.84	16.84
	Male	10.68	4.49	14.65	5.86	12.98	3.46	14.56	4.09	9.61	3.94	62.51	18.41
	t	1.989		2.163		3.372		3.905		3.198		3.398	
	*p*	**0.047**		**0.031**		**0.001**		**0.001**		**0.002**		**0.001**	
Situation of experiencing loss of a relative	Yes	12.42	4,55	16.90	5,73	14.16	3,32	16.29	3,48	11.27	3,63	71.06	17,60
	No	10.63	4,25	14.69	5,88	13.39	3,48	14.99	3,92	9.85	3,77	63.57	17,53
	t	3,586		3,301		1,959		2,978		3,309		3,719	
	*p*	**0.001**		**0.001**		0.051		**0.003**		**0.001**		**0.001**	
Home loss situation	Yes	13.11	4.27	17.90	5.65	14.42	3.51	16.44	3.54	11.63	3.70	73.52	17.42
	No	10.50	4.27	14.49	5.76	13.35	3.39	15.02	3.87	9.82	3.71	63.20	17.27
	t	5.056		4.913		2.589		3.096		4.028		4.932	
	*p*	**0.001**		**0.001**		**0.010**		**0.002**		**0.001**		**0.001**	

Bold values indicate statistically significant differences (*p* < 0.05).

When examining the analysis results regarding the gender variable, it was determined that there was a statistically significant difference between the groups in terms of the sub-factors of the scale and the total scale score.

When the analysis results regarding the variable of experiencing a relative’s loss were examined, it was determined that there was a statistically significant difference between the groups in terms of the sub-factors of the scale, excluding the affective sub-factor, and in terms of the total scale score.

When the analysis results regarding the variable of experiencing home loss were examined, it was determined that there was a statistically significant difference between the groups in terms of the sub-factors of the scale and the total scale score.

## 4 Discussion

This study examined the levels of post-earthquake trauma in athletes and investigated the relationship between being an athlete and traumatic symptoms. The study’s findings showed that athletes had above-average levels of post-earthquake trauma. This result indicates that, although athletes are generally considered a psychologically resilient group, major natural disasters can have significant psychological effects on them. The total score obtained from the Post-Earthquake Trauma Level Assessment Scale was above average. Furthermore, the sub-factors of the scale—affective, cognitive structures, and sleep problems—also had above-average scores. The behavioral problems factor was slightly below average, while the affective factor received an average score. The high differences in the affective, cognitive structure, and sleep problems sub-factors demonstrate that the disaster caused multifaceted psychological effects on athletes. A meta-analysis reviewing 52 studies generally indicated the presence of post-traumatic stress disorder in individuals who experienced earthquakes (Tang et al., 2017). On the other hand, the literature emphasizes that sport is not only an area affected by trauma, but also an important tool for strengthening social solidarity during disasters. Following the Kahramanmaraş earthquake, it was noted that sports organizations, sports administrators, and athletes played an active role in the relief efforts; sport functioned as a unifying force bringing individuals together. It is particularly stated that emotional support is as important as tangible aid for disaster victims. This supports the finding in the present study that athletes had high levels of emotional distress and psychological impact (Türkay et al., 2024).

A study conducted on another sample group that experienced the same earthquakes (February 6, 2023 Kahramanmaraş Earthquakes) as our sample group indicated that the mean overall score on the Post-Earthquake Trauma Level Determination Scale was at a moderate level of trauma. However, the level of physical activity was not found to be a determinant of this. This shows that engaging in sports does not have a calming effect on the level of post-earthquake trauma. This is consistent with our findings (Tosun et al., 2025).

Some studies have also mentioned the need for psychological measures for athletes who have experienced earthquakes. A study on student athletes highlighted that students who experienced earthquakes have a high risk of developing post-traumatic stress disorder (PTSD) and that psychological measures should be taken to address this (Eratlı Şirin et al., 2024). This situation was also determined to be the same in another sample group of student athletes (Atılgan and Öztürk, 2025). Contrary to the data in our study, there are also studies in the literature that indicate that physical activity and exercise can be used as tools for normalizing the level of post-earthquake trauma. A study on a group with high levels of earthquake trauma mentioned the positive effects of exercise in coping with trauma (Çelik et al., 2023). Physical activity has a particularly high healing power for children who have experienced earthquakes (Elçi et al., 2023). Similarly, there are studies supporting the positive effect of regular exercise on post-traumatic stress disorder (Çakır et al., 2025). This supports the idea that physical activity may play a protective role in post-earthquake psychological processes. Indeed, a study conducted on university students after the Kahramanmaraş earthquake, similar to the sample of the current study, reported that the pleasure derived from physical activity was negatively correlated with rumination and was at a higher level in individuals who participated in sports. These findings reveal that physical activity can be an important element in reducing cognitive load and supporting psychological well-being in the post-traumatic period (Güler and Başkara, 2024).

When we examined whether gender was a determinant of the level of trauma after the earthquake, it was observed that female athletes had a higher level of trauma than male athletes. This finding is consistent with previous studies indicating that post-traumatic stress disorder and anxiety levels are higher in women (Gökmen Coşgun, 2022; Çelik et al., 2023; Alpullu and Yılgın, 2024). When the sub-factors of the scale were examined, it was seen that women had higher levels of behavioral problems, emotive limitedness, affective responses, cognitive structures, and sleep problems compared to men. Studies in the literature also emphasize that the negative effects of the earthquake on the cognitive and emotional levels are more pronounced in women (Berkay et al., 2003; Alpullu and Yılgın, 2024). In addition, the age factor is not significant in women, and the same is observed in children. Girls experience more traumatic situations after the earthquake than boys (Ayas, 2005). This situation does not change in adult women either (Liang et al., 2021). This difference observed in the gender variable is thought to be related to genetic and biological factors. Factors such as women being more sensitive to stress hormones and having lower levels of effective coping strategies compared to men cause them to interpret natural disasters more negatively (Zhou et al., 2013). Supporting this, it is thought that women have higher levels of trauma due to their protective instincts and vulnerability tendencies (Atılgan and Öztürk, 2025).

The study determined that individuals who experienced the loss of a relative experienced earthquake trauma at a higher level. Except for the affective sub-factor of the Post-Earthquake Trauma Level Assessment Scale, all sub-factors and the overall mean score of the scale showed that individuals who experienced the loss of a relative experienced higher levels of trauma. Studies indicate that individuals who have experienced the loss of a relative are more likely to have symptoms of post-traumatic stress disorder, depression, and anxiety (Cao et al., 2003; Thienkrua et al., 2006; Fan et al., 2011). Witnessing someone’s death in an earthquake leads to a high incidence of post-traumatic stress disorder and consequently an increase in anxiety levels (Zhou et al., 2013). Furthermore, individuals separated from their families after an earthquake exhibit more trauma symptoms than those who remain with their families (Ayas, 2005). Not only the loss of a relative, but also the loss of a loved one significantly affects the level of trauma (Çakır et al., 2025). In the post-earthquake period, it has been observed that individuals who experienced the earthquake and lost loved ones have a higher level of earthquake knowledge compared to those who did not lose loved ones (Bayraktar et al., 2024). Looking at the literature, we have also found studies showing no significant difference in trauma levels between earthquake victims who lost a loved one and those who did not (Kardaş and Tanhan, 2018; Çelik et al., 2023).

When we examine the trauma levels of athletes who lost their homes as a result of the earthquake, it is seen that they experience more trauma than those who did not lose their homes. Studies have shown that individuals whose homes were destroyed or damaged in the earthquake experience more post-traumatic stress disorder than those who continue to live in their homes. Individuals living in containers, in particular, have higher levels of trauma. Since homelessness leads to both material (heating, shelter, food, etc.) and emotional (loss of memories) losses, the severity of trauma is seen to be higher (Çelik et al., 2023). Severe home damage prevents people from caring for their families. This significantly increases the severity of trauma. Serious social interventions are needed to balance the negative effects of trauma in a positive way (Tang et al., 2017).

In conclusion: Studies in the field of sports show that individuals’ psychological characteristics (e.g., self-confidence and decision-making skills) play an important role in crisis situations (Atılgan and Kaplan, 2022). This supports the idea that in traumatic life events such as earthquakes, psychological processes should be evaluated not only in terms of the level of trauma, but also in conjunction with individual coping and cognitive mechanisms. The negative psychological effects that arise after a disaster can vary depending on individual differences (Tanhan and Kayri, 2013). Furthermore, not every traumatic event will have the same effect on everyone (Gökmen Coşgun, 2022; Atılgan et al., 2025). Some findings indicate that post-earthquake psychological processes are not limited solely to the level of trauma, but are also related to individuals’ coping capacity and psychological resilience. Indeed, a study conducted by Uzun and Atılgan (2026) on teachers reported a significant and negative correlation between post-earthquake trauma and psychological resilience. This suggests that as the level of trauma increases, individuals’ psychological resources may weaken, and consequently, their adaptation processes may be negatively affected. Studies in the literature following the Kahramanmaraş earthquake show that trauma creates strong psychological effects not only at the individual level but also at the collective level. This reveals that post-earthquake psychological processes have a multidimensional structure and that, in addition to individual cognitive processes (e.g., rumination), societal impacts should also be taken into consideration (Kaya Kılıç et al., 2025). It is emphasized that within this multifaceted structure, physical, psychological, and social factors must be considered together; and that sport and physical activity are an important complementary element in this process (Kurt et al., 2024; Avan and Parlakyıldız, 2024). In this context, the increase in cognitive processes such as rumination and the decrease in protective factors such as physical activity offer an important holistic approach to explaining post-earthquake psychological well-being. Individuals who have experienced a major earthquake, whether athletes or not, may experience negative psychological states. Exercise and physical activity have a significant effect in reducing the negative effects of earthquake trauma. However, in some cases, exercise and physical activity may also be insufficient (Thorpe, 2015). Women are more affected than men in traumatic situations. Post-traumatic stress disorder is experienced at a very high level in earthquake victims who have lost a relative or loved one. Individuals who have lost their homes in the earthquake experience more severe traumatic processes because they cannot meet their needs for shelter, heating, and security.

### 4.1 Limitations

This research has several limitations. First, the study has a cross-sectional design. Therefore, the findings reflect only the situation within a specific time period, and it is not possible to establish causal relationships. Second, research data were collected through self-report scales. This suggests that participants may have been influenced by factors such as social desirability bias or recall bias. Third, the sample consists only of licensed athletes living in 11 provinces affected by the February 6, 2023 Kahramanmaraş earthquake. Therefore, generalization of the findings to the entire athlete population or to different cultural and geographical contexts is limited. Fourth, snowball sampling, a non-probability sampling method, was used in sample selection. This may limit the representativeness of the sample. The use of snowball sampling can increase the likelihood of selecting participants from similar social backgrounds, thus increasing the risk of sampling bias and limiting the generalizability of the findings. However, this method was preferred due to the difficulty of reaching athletes located in the disaster area after the earthquake. It is recommended that future studies be conducted with larger sample sizes and using probabilistic sampling methods. Finally, this study only considered certain sociodemographic variables (gender, loss of a loved one, and loss of home).

## Data Availability

The raw data supporting the conclusions of this article will be made available by the authors, without undue reservation.
